# Can Peripheral Skin Perfusion Be Used to Assess Organ Perfusion and Guide Resuscitation Interventions?

**DOI:** 10.3389/fmed.2020.00291

**Published:** 2020-06-23

**Authors:** Jan Bakker, Glenn Hernandez

**Affiliations:** ^1^Department of Pulmonary and Critical Care, Bellevue Hospital, NYU Langone, New York, NY, United States; ^2^Division of Pulmonary and Critical Care Medicine, Columbia University Medical Center, New York, NY, United States; ^3^Department of Intensive Care Adults, Erasmus MC University Medical Center, Rotterdam, Netherlands; ^4^Pontificia Universidad Católica de Chile, Department of Intensive Care, Santiago, Chile

**Keywords:** capillary refill time (CRT), shock, treatment, infection, sepsis

## Abstract

Although the definition of septic shock is straightforward, the physiological response to inadequate hemodynamics in patients with septic shock is variable. Therefore, the clinical recognition is limited not only by the patient's response but also by the clinical parameters we can use at the bedside. In this short overview we will argue that the state of the peripheral perfusion can help to identify and to treat patients with septic shock.

## Introduction

Recently a group of experts defined shock as “A life-threatening, generalized form of acute circulatory failure associated with inadequate oxygen utilization by the cells. It is a state in which the circulation is unable to deliver sufficient oxygen to meet the demands of the tissues, resulting in cellular dysfunction” (1). In the context of an active infection this would be regarded as septic shock. Although this is a very straightforward definition the application to the bedside is problematic at best. In addition, the new definition of septic shock using symptoms yielding the best ability to predict mortality: a mean arterial pressure requiring administration of a vasopressor to maintain a given level and an increased lactate level, may therefore not necessarily provide much clinical guidance to the treatment of septic shock (2).

The identification of an inadequate delivery of sufficient oxygen to the tissues in relation to their unknown demand is a complex process that has no readily available parameters that identify the core of this definition at the bedside. That is why we use clinical surrogates aimed at restoring tissue oxygenation during the resuscitation of patients. In the consensus, the parameters advocated are serum lactate concentration next to parameters of microcirculatory perfusion like mottled skin, acrocyanosis, prolonged capillary refill time and an increased central-to-toe temperature gradient (1). Where increased lactate levels are thought to reflect the imbalance between oxygen delivery to the tissues and oxygen consumption by the tissues, the microcirculatory parameters likely reflect the physiologic response to acute changes in global blood flow shutting down perfusion of non-vital organs (e.g., skin). Therefore, here we will focus on the clinical use of this perfusion parameter in the clinical practice of early septic shock resuscitation.

## Basic (PATHO)Physiologic Principles

The perfusion of the skin is mainly used for thermoregulation and is mostly under myogenic control (sympathetic nervous system) rather than metabolic control (3). When blood pressure drops the associated sympathetic response is vasoconstriction resulting in a decrease in the diameter of the arterioles thereby reducing blood flow significantly (4). A 50% decrease in the diameter will decrease blood flow by 94%. This contrasts significantly to the effect of vasodilation where a 50% increase in the diameter increases blood flow by 400% (4). Autoregulation can be seen as the vasodilatory response to the initial vasoconstriction in order to maintain organ blood flow (5). However, as skin blood flow is hardly autoregulated this primary effect (decreased blood flow) persists until the cause of the sympathetic activation has been resolved.

In experimental models of decreased cardiac output due to simulated hypovolemia (lower body negative pressure) indicators of peripheral perfusion indicate hypoperfusion of the skin (6–8). In experimental models of decreases in cardiac output (CO) either due to tamponade or endotoxin infusion, sublingual microcirculatory perfusion decreases while its restored following resuscitation (removal of tamponade and fluids) (9). In simulated hypovolemia in healthy volunteers the decrease in CO is also related to decreased sublingual microcirculatory perfusion (10). In clinical conditions, impaired peripheral perfusion also marks circulatory shock in many circumstances including septic shock (11–14).

## Relationship To Outcome and Treatment

Many studies in clinical practice have shown parameters of peripheral perfusion being related to morbidity and mortality in different contexts. Originally, the capillary refill time (CRT) was used in traumatic shock as an element in severity/outcome prediction (15). Many later studies showed that any parameter associated with impaired peripheral perfusion (near infrared, CRT, subjective temperature, skin temperature difference etc.) was related to outcome in a variety of clinical conditions (16).

Also, during the resuscitation of patients with circulatory dysfunction, an abnormal indicator of peripheral perfusion is associated with poor outcome. In a study in the emergency department in patients with sepsis, Lara et al. showed that an abnormal CRT after initial fluid resuscitation was associated with an almost 6 times higher mortality when compared to patients having a normal CRT at that time (17). Lima et al. showed that abnormal peripheral perfusion following initial resuscitation in patients with circulatory dysfunction including sepsis was also associated with increased morbidity (18, 19). In addition, increased lactate levels in the presence of indicators of abnormal perfusion, including CRT, are associated with significantly higher morbidity and mortality in septic shock patients (20). Parameters of peripheral perfusion respond rapidly to treatments aimed to restore tissue blood flow (fluid resuscitation, vasodilators, multimodal interventions) (21, 22). Finally, a small hypothesis generating study showed that fluid resuscitation can be mitigated in patients with normal peripheral perfusion resulting in decreased morbidity (23).

With all this evidence, linking abnormal peripheral perfusion to outcome parameters and the effect of treatment aimed to improve tissue perfusion and its effect on parameters of peripheral perfusion and outcome in mind, the ANDROMEDA-Shock study was designed (24). In this study, the clinical endpoint of a normal CRT (≤ 3 s) was used to resuscitate patients in the early phase of septic shock (protocol started within 4 h of diagnosis). Given the recommendation of the Surviving Sepsis Guideline and the results of a recent meta-analysis, clearance of lactate or normalization of lactate levels was used as the resuscitation endpoint in the control group (25, 26). The main elements of the protocol were the sequential steps starting with fluid challenges, followed by vasoactive-related interventions when endpoints were not met. Although the difference in the primary outcome, day 28 mortality, just missed statistical significance (34.9 vs. 43.4%; *p* = 0.06), secondary outcomes suggested decreased morbidity in the protocol group (27). In addition, a reanalysis of the data using the Bayesian approach showed that even when using a primer where CRT guided resuscitation would result in a 100% increase in mortality compared to what was found in the actual results of the study, the likelihood patients would benefit from a CRT guided approach was more the 90% (28).

Brunauer et al. (29) recently showed that abdominal organ perfusion was related to CRT in patients with septic shock. In the light of these findings a resuscitation aiming to normalize CRT seems to address a possible clinically relevant problem and is underscored by the faster resolution of organ failure in the CRT guided resuscitation group (27). Also, the results of the study underscore the hypothesis generating small randomized trial in septic shock patients showing that withholding fluid resuscitation in patients with normal peripheral perfusion resulted in a decrease in organ failure (23) as also in the ANDROMEDA-Shock study less fluids were used in the CRT guided resuscitation group.

## The Future Use of Peripheral Perfusion Guided Resuscitation

As a target of early resuscitation, its strong connection to physiologic parameters and interventions (17, 29, 30), CRT seems to signal tissue reperfusion in septic shock patients. Therefore, a rapid positive response to initial treatment might relate to a status of hemodynamic coherence (31). This is underscored by the large difference in mortality between responders (patients that normalize their CRT after fluid resuscitation) and non-responders (9–23 vs. 45–55% respectively) in different studies (17, 27, 32). In addition, changes in CRT following treatment track changes in abdominal organ perfusion (29), are responding rapidly to treatment (21) and finally are associated with less organ failure and a faster improvement in organ failure (19, 27).

Therefore, a septic shock patient presenting with normal peripheral perfusion or a rapid normalization of abnormal peripheral perfusion parameters might reflect a different clinical profile that could benefit from a more restricted resuscitation strategy (23, 27, 28). Where a patient with a loss of hemodynamic coherence could warrant a different approach (31).

The response of the peripheral perfusion to an intervention aimed to improve tissue blood flow could thus represent a hemodynamic coherence test where a parallel improvement in parameters used to assess the adequacy of tissue perfusion may be expected in patients that correct their abnormal peripheral perfusion parameter (33).

Although promising, these hypotheses need significant future work to provide a solid place for peripheral perfusion parameters in the resuscitation of septic shock patients.

## Author Contributions

All authors listed have made a substantial, direct and intellectual contribution to the work, and approved it for publication.

## Conflict of Interest

The authors declare that the research was conducted in the absence of any commercial or financial relationships that could be construed as a potential conflict of interest.
